# Induction of neutralizing antibody responses by AAV5-based vaccine for respiratory syncytial virus in mice

**DOI:** 10.3389/fimmu.2024.1451433

**Published:** 2024-10-14

**Authors:** Gangyuan Ma, Zeping Xu, Chinyu Li, Feng Zhou, Bobo Hu, Junwei Guo, Changwen Ke, Liqing Chen, Guilin Zhang, Hungyan Lau, Hudan Pan, Xixin Chen, Runze Li, Liang Liu

**Affiliations:** ^1^ Guangzhou National Laboratory, Guangzhou, China; ^2^ Guangzhou Medical University, Guangzhou, China; ^3^ Guangdong Keguanda Pharmaceutical Technology Co., Ltd, Guangzhou, China; ^4^ Guangdong Provincial Center for Disease Control and Prevention, Guangzhou, China; ^5^ State Key Laboratory of Traditional Chinese Medicine Syndrome, The Second Affiliated Hospital of Guangzhou University of Chinese Medicine (Guangdong Provincial Hospital of Chinese Medicine), Guangzhou, China; ^6^ Chinese Medicine Guangdong Laboratory, Hengqin, China

**Keywords:** RSV vaccine, rAAV5 vector, pre-fusion protein, humoral immunity, cellular immunity

## Abstract

**Introduction:**

Respiratory Syncytial Virus (RSV) is a significant cause of respiratory illnesses worldwide, particularly in infants and elderly individuals. Despite the burden RSV imposes, effective preventive measures are limited. The research application of adeno-associated virus (AAV) in vaccine platforms has been expanding, and its potential in prevention and treatment has garnered much attention.

**Methods:**

In this study, we explored the potential application of a recombinant adeno-associated virus 5 (rAAV5) vector-based RSV vaccine, focusing on the expression of the pre-fusion (Pre-F) protein structure. Through intramuscular immunization in mice. The immunogenicity of the vaccine was evaluated in Balb/c mice immunized intramuscularly and intranasal, respectively.

**Results:**

The rAAV5-RSV-Fm vaccine demonstrated positive humoral and induced antibody titers against RSV strains A and B for up to 120 days post-immunization. Notably, intranasal administration also elicited protective antibodies. Characterization studies confirmed the ability of the vac-cine to express the Pre-F protein and its superior immunogenicity compared to that of full-length F protein.

**Conclusion:**

These findings underscore the potential application of rAAV5 vector platforms in RSV vaccine development and further investigation into their protective efficacy is warranted.

## 1 Introduction

Respiratory syncytial virus (RSV) is a formidable adversary in the realm of respiratory pathogens as RSV poses a significant global health burden, particularly among vulnerable demographics such as infants and elderly individuals. This single-stranded RNA virus, belonging to the family Pneumoviridae, is notorious for causing upper and lower respiratory tract infections, often leading to severe complications such as bronchiolitis and pneumonia. Among children under five years of age, RSV is one of the primary causes of hospitalization, with infants under six months being especially susceptible (1, 2). Furthermore, the elderly population, including those aged 60 years and older, faces a RSV burden comparable to that posed by seasonal influenza in adults over 65 years old. By the year 2022, the global incidence of severe RSV infections among individuals aged 65 and above is estimated to reach a staggering 8.6 million (3, 4).

Despite its significant impact on public health, prophylactic measures against RSV that are effective over long durations remain elusive. Currently, therapeutic options are largely limited to supportive care, leaving high-risk populations with minimal options beyond symptomatic relief. Thus, the development of preventive strategies is paramount for mitigating the burden of RSV-related morbidity and mortality. Traditional methods of acquiring immunity through natural infection fail to confer long-term protection against RSV due to the ability of RSV to evade immune memory responses (5), thus necessitating the pursuit of active preventive measures such as vaccination.

To date, the arsenal of RSV vaccines remains sparse, with only two prophylactic products receiving approval from the US Food and Drug Administration (FDA) for individuals aged 60 and above as of 2023: Arexvy from GSK and ABRYSVO from Pfizer (6–8). These vaccines, which are based on recombinant protein constructs, represent important advancements in RSV prevention. However, further innovation is imperative to address the pressing need for more efficacious and broadly protective vaccine formulations.

Central to the development of effective RSV vaccines are virus surface glycoproteins, particularly the fusion (F) and attachment (G) proteins, which serve as primary targets for host immune responses. The F protein, in particular, holds promise as a vaccine antigen due to its essential role in mediating viral fusion and its relatively conserved nature across RSV strains (9–11). Notably, the F protein exists in two conformational states—pre-fusion (Pre F) and post-fusion (Post F)—with the former being the preferred target for vaccine-induced immunity due to the exposure of critical neutralizing epitopes (12, 13). Leveraging this knowledge, recent vaccine candidates have focused on stabilizing the Pre F conformation to improve immunogenicity and efficacy (14–16).

In parallel, the emergence of gene therapy vectors, particularly recombinant adeno-associated virus (rAAV), has revolutionized vaccine development by offering a versatile platform for antigen delivery. rAAV vectors, which are derived from non-pathogenic parvoviruses, boast attributes such as low immunogenicity, broad tissue tropism, and safety, making them ideal candidates for vaccine delivery (17–20). Previous research has demonstrated the potential of rAAV vectors in the development of vaccines against infectious diseases, including the successful application of an rAAV5-based vaccine against SARS-CoV-2 (21).

Based on this foundation, in the present study, we explored the feasibility of utilizing an rAAV5 vector platform for the development of an RSV vaccine. Specifically, we engineered rAAV5 vectors encoding the wild type F protein (RSV-F), Pre-F protein (RSV-Fm) and wild type G protein (RSV-G) of RSV respectively, then evaluated their immunogenicity in a murine model. By assessing both of the humoral and cellular immune responses elicited by intramuscular and intranasal immunization routes identified the rAAV5-RSV-Pre-F vaccine as the optimal design with positive immunogenicity. We aimed to evaluate the potential application of rAAV5-based RSV vaccines to confer effective and durable immunity against this pervasive respiratory pathogen. In summary, this study represents a step forward in the pursuit of innovative vaccine strategies against RSV and underscores the potential application of rAAV-based platforms for combating infectious diseases.

## 2 Materials and methods

### 2.1 Cells and virus

Human embryonic kidney cells 293T (HEK293T cells) (ATCC) were cultured in Dulbecco’s modified Eagle’s medium (DMEM, Gibco, USA) supplemented with 10% fetal bovine serum (FBS). Hep-2 cells, live RSV subtype A (strain Long) and subtype B (strain 9320) virus (Hainan Tropical Infectious Diseases Biobank) was obtained from the Guangdong Provincial Center for Disease Control.

### 2.2 Animals

Six- to eight-week-old specific pathogen-free female BALB/c mice were purchased from Bestest. Animal research was approved by the Guangzhou National Laboratory Animal Care and Use Committee (Ethics number: GZLAB-AUCP-2022-10-A08). Mice were housed in a temperature-controlled environment with a 12-h light-dark cycle and provided commercial mouse food and water ad libitum.

### 2.3 Immunization protocols

In the first batch, mice (5 animals per group) were immunized with rAAV5-based vaccines or PBS (control group) via intramuscular (i.m.) injection in the hind leg on day 0. In the second batch, mice (10 animals per group) were inoculated intranasally by slowly pipetting a volume of 20 µl into one nostril containing the final dose of vaccine. Sera were collected at various time points after immunization for analysis. PBMCs were isolated by split-red method and analyzed by flow cytometry.

### 2.4 Construction of AAV5-based vaccine plasmids

Three AAV-based vaccine candidates were tested: rAAV-RSV-F, rAAV-RSV-F-m, and rAAV-RSV-G (GenBank: ACO83301.1 and GenBank: URP22622.1). rAAV-RSV-F is an AAV5 vector that expresses the codon-optimized, full-length RSV F protein under the control of a CMV promoter. rAAV-RSV-Fm has two mutated sites (N67I and S215P) based on rAAV-RSV-F (22). rAAV-RSV-G is an AAV5 vector that expresses the codon-optimized, full-length RSV G protein under the control of a CAG promoter. Each vaccine construct included additional regulatory elements: a woodchuck hepatitis virus posttranscriptional regulatory element (WPRE) and a bovine growth hormone polyadenylation signal (poly-A). Western blots were using to verify the expression of the target antigen of the plasmid. The target gene plasmid was transfected into 293T cells with PEI (Polysciences, 24765-1) to express the target protein. Proteins were separated by electrophoresis in SurePAGE 4%–20% polyacrylamide gels (Genscript: M00655) and then transferred to poly-vinylidene fluoride (PVDF) membranes. RSV-F (A2) antibody (1:2000 Sino Biological: 11049-R302) and anti-HRSV-A G/major surface glycoprotein G antibody (1:5000 3D3, Antibody system: RVV08502) followed by peroxidase-conjugated anti-rabbit (Proteintech: SA00001-2), or anti-human secondary antibodies (Jackson Immuno Research: 109-035-003), respectively (1:10,000) were used for it.

### 2.5 Production and purification of the recombinant AAV vectors

The three plasmids (AAV5-based vaccine plasmids, pAAV-RC and pHelper) were co-transfected into 293T cells using PEI transfection reagent for packaging the recombinant AAV vector. The transfected cells were harvested 72h after transfection. Cells were treated with Lysis buffer (50mM Tris-HCl, 150mM NaCl, 2mM MgCl2, PH=8.0) and then subjected to 4 freeze–thaw cycles. Broad-spectrum unrestricted nuclease was added and the supernatants were collected by centrifugation at the end of incubation at 37°C. At the same time, PEG8000 was added to a final concentration of 8%(w/v), subsequent precipitation at 4°C overnight. The supernatants were removed by centrifugation the next day. At the end of centrifugation, the precipitate was resuspended using 10ml PBS and ultracentrifugation with iodixanol density gradient. The titers of the rAAV5 vaccine were determined by digital PCR (Bio-rad ddPCR Supermix for Probes:186-3024) with the following primer pairs. Primer pair for WPRE: forward 5’-ACAATTCCGTGGTGTTGTCGG-3′, reverse 5′-AGGAAGGTCCGCTGGATTGA-3′ and TaqMan probe FAM- ACCTGGAT-TCTGCGCGGGA-BHQ1.The purity of rAAV5 was confirmed by Coomassie brilliant blue staining. SEC-HPLC method and SRT SEC-500 column were used to detect the aggregates of rAAV5 vector. For the detection of the empty AAV capsid, the AEC-HPLC method and BIA/CIMac™ AAV empty/full - 0.1 Analytical Column were used to separate the empty capsid from the intact AAV5 vector using the AAV empty/full capsid analytical column (23).

### 2.6 *In vitro* infection and antigen expression by flow cytometry

The surface expression of F protein on infected cells was measured by immunofluorescence using flow cytometry. Cultured 293T cells were transduced with the rAAV5 vaccine, and 1×10^11^vg of the virus were added. Forty-eight hours after AAV infection, the cells were harvested and then stained with Alexa488-conjugated D25 (Antibody system: RVV02809), 4D7(Antibody system: RVV02817) and AM14 (Antibody system: RVV02801). rAAV5-RSV-G vaccine using Alexa488-conjugated 3D3 (Antibody system: RVV08502). The cells were then analyzed by flow cytometry. Binding of the D25 anti-body indicated the presence of the antigenic site Φ on the pre-fusion F protein, binding of 4D7 indicated a post-fusion RSV F or intermediate conformation, and binding of AM14 indicated a trimeric form of pre-fusion RSV F. Binding of 3D3 indicated correct expression of RSV-G protein (24).

### 2.7 Serum ELISA

The RSV-specific IgG antibodies were analyzed using an enzyme-linked immuno-sorbent assay (ELISA). Briefly, 96-well microplates were coated with RSV-F Protein (ECD, His-Tag sino biological:11049-V08B) or RSV-G Protein (ECD, His-Tag sino bio-logical:40041-V08H) (0.5 µg/mL) and incubated overnight at 4°C. The next day, the plates were washed with PBST (PBS with 0.1% Tween 20), followed by a blocking step with 2% skim milk for 1 h at room temperature. Serially diluted mouse serum was added to the wells, followed by incubation for 90 min at 37°C. After three washes, horseradish peroxidase (HRP)-conjugated goat anti-mouse IgG (1:5000 Proteintech: SA00001-1) was added to the wells, followed by incubation for 1 h at 37°C. After washing, the substrate 3,3,5,5-tetramethylbenzidine (Invitrogen: 00-4201-56) was added to the plates, and the reaction was stopped by adding 1 M H2SO4. The absorbance at 450 nm was measured by an ELISA plate reader (Biotec, Hercules, CA, USA). The endpoint serum dilution was calculated with a curve to fit the analysis of optical density (OD) values for serially diluted sera with a cut-off value of negative control.

### 2.8 Flow cytometry

Flow cytometry was used to determine the level of immune T lymphocytes in the peripheral blood of mice after immunization. Anticoagulated mouse blood samples were collected, and flow sample preparation was performed after adding red blood cell lysate. The cells were then stained with anti-CD3-Pe-Cy7 (biolegend: 100220), anti-CD4-PerCp (biolegend: 100432), and anti-CD8-FITC (biolegend: 100706). The cells were then permeabilized with Cytofix/Cytoperm and subsequently intracellularly stained with anti-IFN-γ-PE (biolegend: 505808). The percentage of CD3+CD4+ and CD3+CD8+ T cells expressing IFN-γ was quantified by flow cytometry using Agilent Novocyte Advanteon. Analysis of flow cytometric data was performed in FlowJo software version 9.6.1.

### 2.9 Virus neutralization assay

Hep-2 cells were plated at a density of 2×10^4^ cells/well in a 96-well plate and grown at 37°C overnight. Mouse sera were heated at 56°C for 30 min. Serial 4-fold dilutions (1:4–1:1024) of mouse sera were separately mixed with 100 TCID50 (50% tissue culture infective dose) of live RSV subtype A (strain Long) and subtype B (strain 9320) virus (Hainan Tropical Infectious Diseases Biobank), then incubated at 37°C for 2 h. The cytopathic effect (CPE) was observed daily and recorded after 7 days. Neutralizing titers of mouse sera that suppressed 50% of the CPE were calculated by using the Reed–Muench method.

### 2.10 Statistical analysis

All values are presented as the mean ± SD. For the mouse studies, ANOVA was used for statistical comparisons between groups. IgG titer data were log10-transformed. Statistical significance for IgG titer and IFN-γ+ cell (%) data was calculated using two-way ANOVA with Dunnett’s multiple comparisons. As for neutralizing antibody data, one-way ANOVA was used if the data passed the normality test; otherwise, the Kruskal-Wallis rank-sum test was applied. Statistical analyses were performed using GraphPad Prism 8 (GraphPad Software Inc., San Diego, CA, USA). The difference between groups is considered statistically significant when p values < 0.05.

## 3 Results

### 3.1 Design and characterization of the rAAV5-RSV-F, rAAV5-RSV-Fm, and rAAV5-RSV-G vaccines

**Figure 1:**
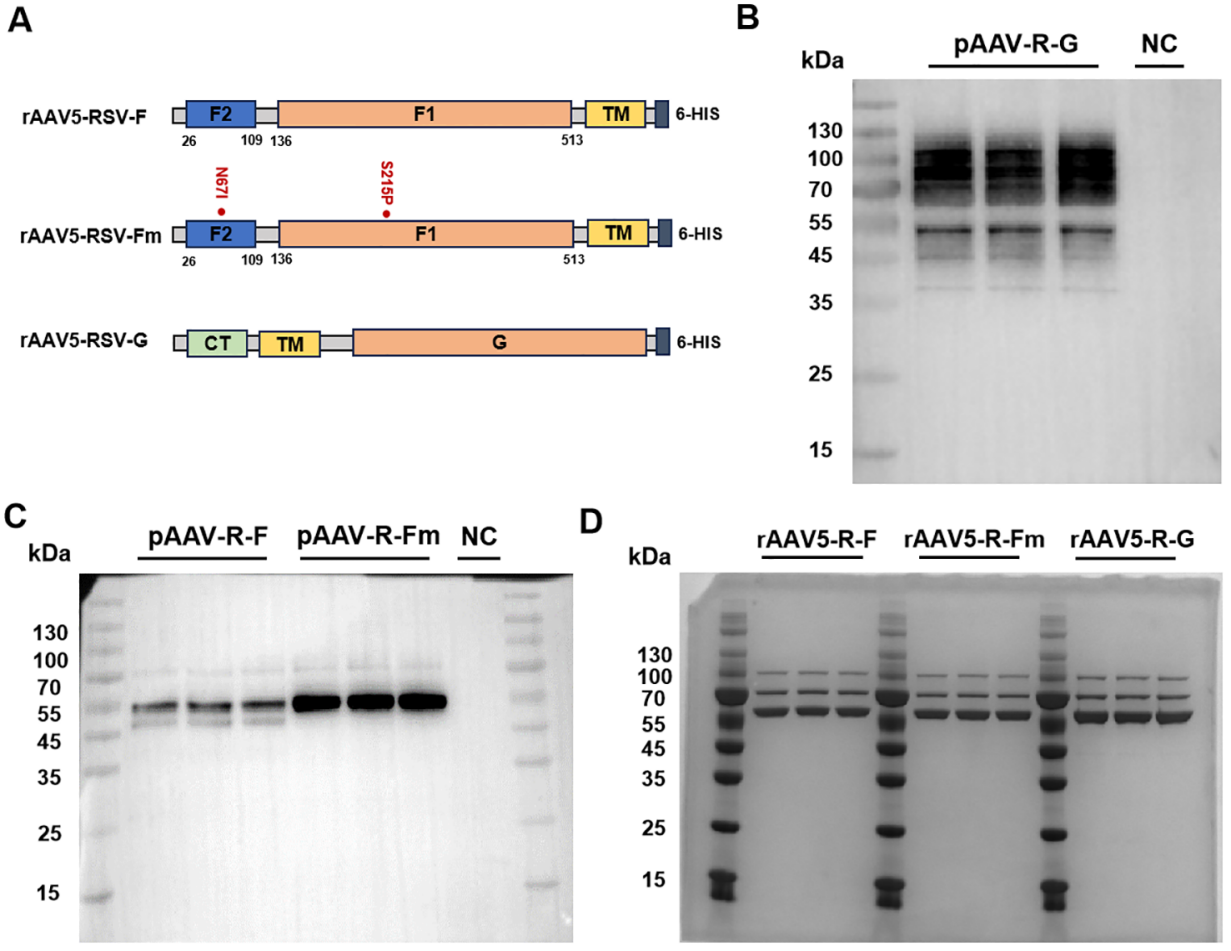
Design and characterization of the rAAV5-RSV-F, rAAV5-RSV-Fm, and rAAV5-RSV-G vaccines. (26-109: F2 subunit; 109-136: P27; 136-513: F1 subunit; CT, cytoplasmic tail; TM, trans-membrane domain). Genetic structure of the target gene plasmids in the rAAV5-RSV-F, rAAV5-RSV-Fm, and rAAV5-RSV-G vaccines **(A)**. Plasmid expression of the target antigen was experimentally validated by Western blotting (F protein and Fm Protein ~ 55kDa, G protein ~90kDa) **(B, C)**. The purity of the rAAV vaccines was verified by coomassie (VP1~87kDa, VP2~73kDa, VP3~62kDa) **(D)**.

The rAAV5-RSV-F vaccine was constructed to express the full-length wild-type F protein (wt F protein), while the rAAV5-RSV-Fm vaccine was designed to produce the pre-fusion (Pre-F) protein by introducing stabilizing mutations (N67I and S215P). It plays a role in stabilizing the Pre-F structure by preventing the formation of long helical strands in the RR1 structural region of the RSV-F protein and stabilizing the apex (22). The rAAV5-RSV-G vaccine encoded the full-length wild-type G protein (**Figure 1A**). As shown by Western blot results, the protein expressed by the target gene plasmid of the rAAV5-RSV-G vaccine correctly bound and was tagged by the anti-HRSV-A G/major surface glycoprotein G antibody (3D3, Antibody system) (**Figure 1B**). The F proteins expressed after transfection of the rAAV5-RSV-F and rAAV5-RSV-Fm target gene plasmids were characterized by Western blotting. The proteins expressed by the rAAV5-RSV-F and rAAV5-RSV-Fm target gene plasmids correctly bound to the RSV-F (A2) antibody (Sino Biological:11049-R302) (**Figure 1C**). The G protein is a transmembrane glycoprotein. The ectodomain is post translationally modified by 4-5 N-linked glycans and 30-40 O-linked glycans, which account for ~60% of the molecular weight of the mature glycoprotein (25, 26), and has many sites of glycosylation, hence the apparent band tailing.

Three plasmid transfection methods were adopted to improve rAAV packaging; iodixanol was used to purify the rAAV, and virus titers were determined by ddPCR. The purity of the capsid protein directly reflected protein impurity. SDS–PAGE combined with Coomassie brilliant blue staining is the most intuitive method for the detection of impure AAV proteins. This method can be used to not only identify AAV but also determine its purity by observing the number of protein staining bands (usually three bands for the VP1, VP2, and VP3 proteins, and sometimes the AAP protein). The ratio of VP1: VP2: VP3 was 1:1:10 (**Figure 1D**). The aggregates of the rAAV5-RSV-F, rAAV5-RSV-Fm and rAAV5-RSV-G vaccines were 2.05%, 1.3% and 1.92%, respectively. And the percentage of empty capsid of the three rAAV5-RSV vaccines were 3.36%, 1.96% and 3.7%, respectively, while the industry standard is less than 30% (27). Both were indicating that the rAAV5 viral vector platform could produce highly purified rAAV vaccines (**Supplementary Figures S1**, **S2**).

### 3.2 Recognition of conformational regions of F protein in the rAAV5-RSV-Fm vaccine

**Figure 2:**
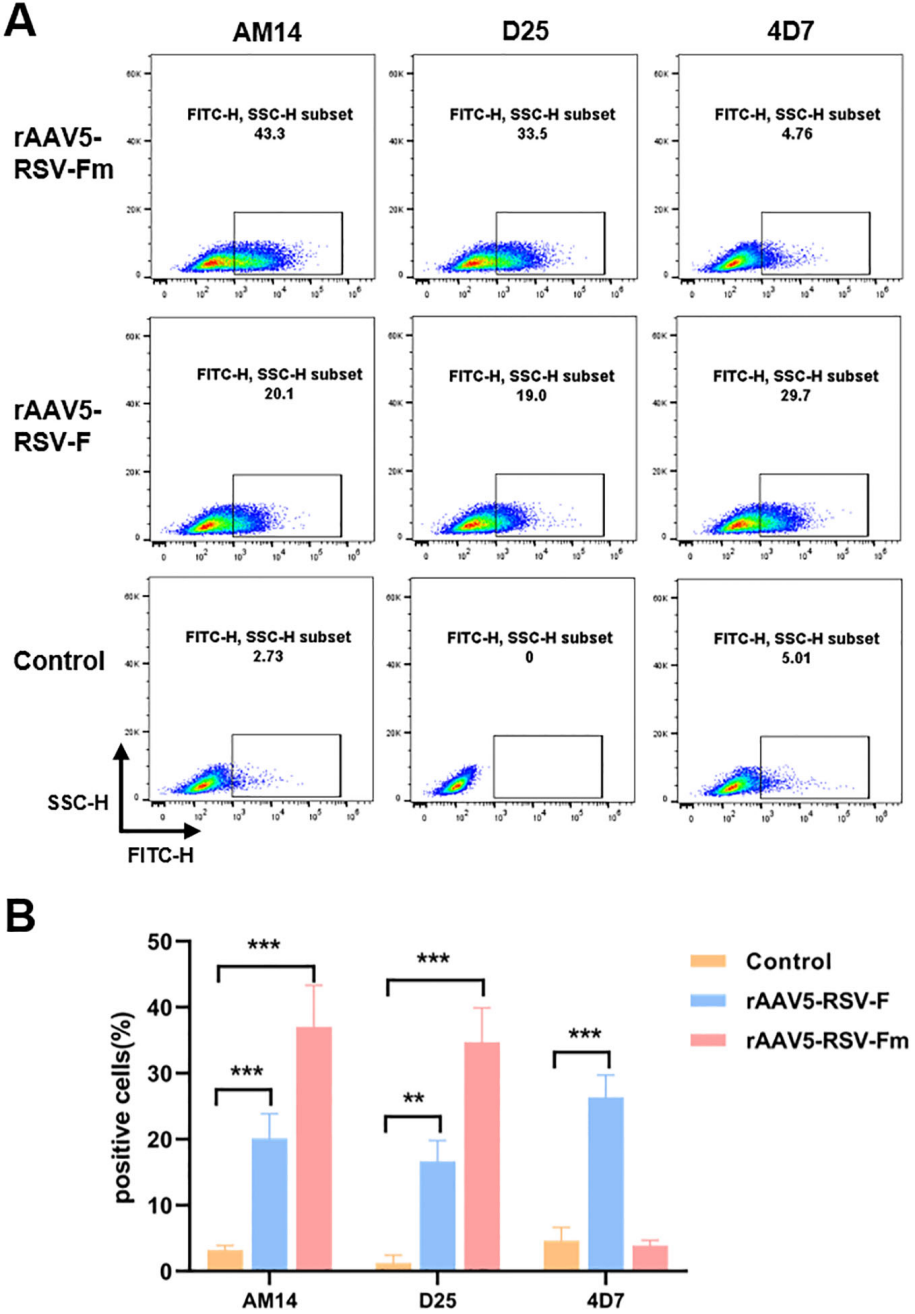
The percentage of cells transfected with the rAAV5-RSV-F and rAAV5-RSV-Fm vaccines that expressed membrane-associated forms of the RSV F protein bound by D25, 4D7, or AM14 was determined **(A, B)**. (n=3) Statistical significance was calculated via two-way ANOVA with Dunnett’s multiple comparisons. P values were adjusted for multiple comparisons. **p < 0.01, ***p < 0.001.

Due to the challenges in RSV Pre-F expression and purification, many studies have aimed to increase Pre-F expression in different cells or its stability while purifying the Pre-F antigen structure. This study is the first to report the expression of the Pre-F anti-gen through the rAAV vector. The constructed rAAV5-RSV-F and rAAV5-RSV-Fm vaccines were used to infect 293T cells *in vitro* for protein characterization. The conformation of the transmembrane form of RSV F was assessed using flow cytometry. Infected cells were stained with conformational indicator antibodies (D25, 4D7, and AM14) and analyzed by flow cytometry. The D25 antibody bound with the antigen site Φ of the pre-F protein (28), while the binding of AM14 indicates the trimeric form of pre-fusion RSV F (22). The binding of 4D7 confirms the existence of post-fusion RSV F (29, 30). These results showed that the binding rates of rAAV5-RSV-F to the 4D7, D25 and AM14 antibodies were 29.7%, 20.1% and 19%, respectively, in rAAV5-RSV-F-infected cells, suggesting that the rAAV5-RSV-F vaccine mainly expressed post-fusion RSV F or intermediate conformation protein. However, the binding rate of the rAAV5-RSV-Fm protein to the 4D7 antibody was only 4.76%, indicating that the rAAV5-RSV-Fm vaccine had little or no effect on the F protein structure in cells infected with the rAAV5-RSV-Fm vaccine. The D25 antibody binding rate was 33.5%, and the AM14 antibody binding rate was 43.3%, which indicates that the rAAV5-RSV-Fm vaccine can successfully express the Pre-F trimer protein structure (**Figure 2**). These results showed that the rAAV vector can be used to successfully induce pre-fusion RSV F expression. The rAAV5-RSV-G vaccine has also been validated for its ability to accurately express the RSV-G protein. (**Supplementary Figure S3**).

### 3.3 The rAAV5-RSV-Fm vaccine induced an effective antibody response in BALB/c mice

**Figure 3:**
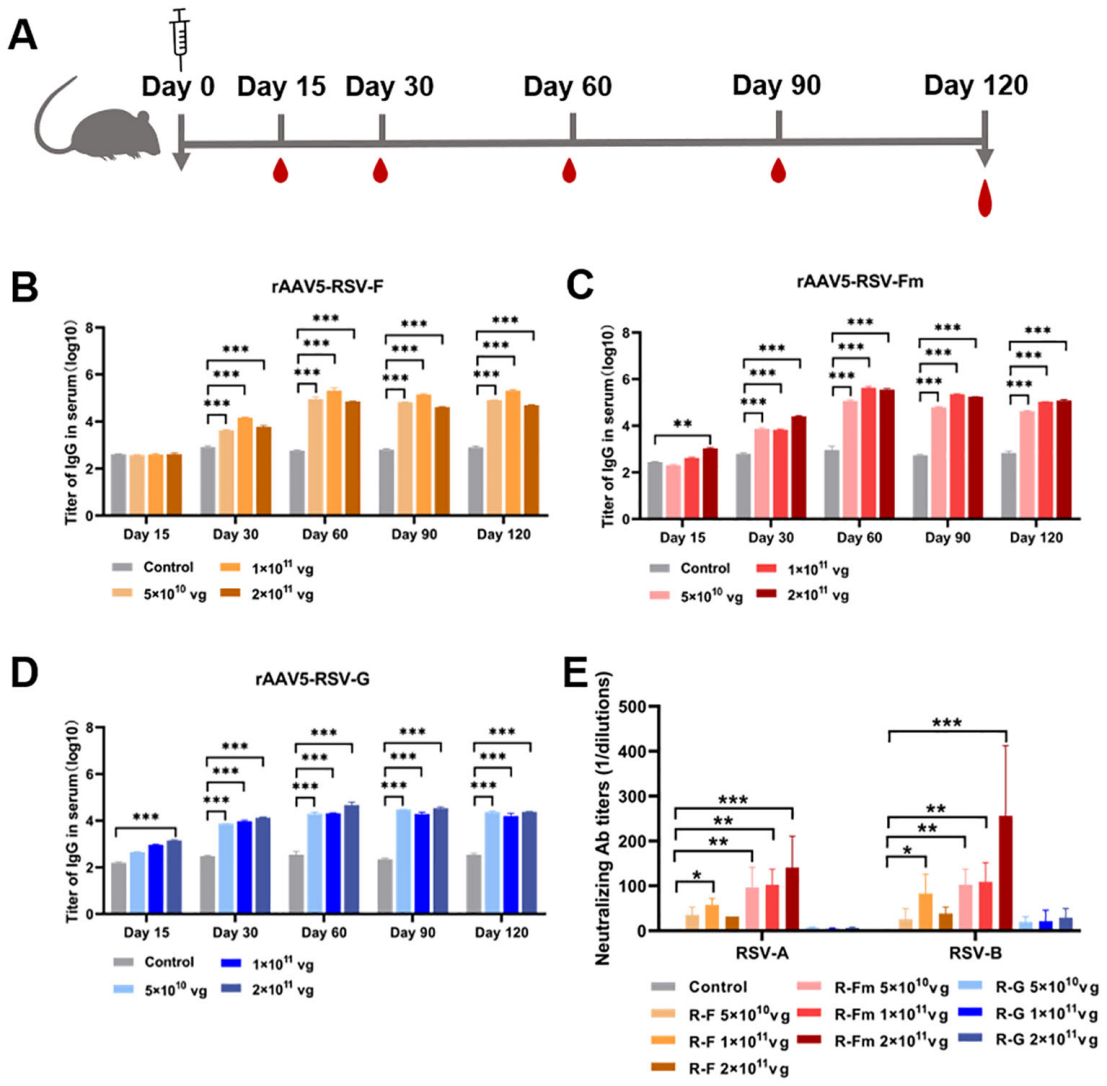
Serum RSV-specific and neutralizing antibody titers induced by recombinant vaccines. **(B–D)** The serum IgG titer 15, 30, 60, 90 and 120 days after immunization was determined by ELISA. **(E)** Neutralizing activity against RSV subtypes **(A, B)** was assessed at day 120 after immunization; the data are presented as the mean ± SD of antibody titer. (n = 5 per group). Statistical significance was calculated via two-way ANOVA with Dunnett’s multiple comparisons **(B–D)**. P values were adjusted for multiple comparisons. Statistical significance was calculated via Kruskal-Wallis rank-sum test **(E)**. *p < 0.05, **p < 0.01, ***p < 0.001.

To confirm whether the rAAV-RSV vaccine could effectively induce specific anti-bodies against RSV, BALB/c mice were immunized with different doses of the rAAV5-RSV-F, rAAV5-RSV-Fm and rAAV5-RSV-G vaccines by intramuscular injection, while the control group was treated with PBS. Mouse sera were collected 15, 60, 90 and 120 days after immunization for IgG analysis (**Figure 3A**). IgG is the most abundant anti-body produced by the body and is the most important for defending against foreign antigen infection. rAAV5-RSV-Fm and rAAV5-RSV-G induced a humoral immune response in mice as early as 15 days after immunization in a dose-dependent manner. The antibody titer of rAAV5-RSV-Fm and rAAV5-RSV-G in the 2×10^11^vg group exhibited a significant elevation compared to the control group, respectively, on day 15. As time progressed to day 60, the serum antibody titer in rAAV5-RSV-F or rAAV5-RSV-Fm immunized mice reached nearly 300000. The conversion to logarithms is close to 5.4, which was monitored and remained stable until day 120, at which point the immune effect was better than that of the rAAV5-RSV-G vaccine (**Figures 3B–D**).

Consistent with this result, we analyzed neutralizing antibodies 60, 90 and 120 days after immunization. The results showed that the sera from rAAV5-RSV-F- and rAAV5-RSV-Fm-immunized mice neutralized both subtype A and subtype B of live RSV. The sera of mice immunized with rAAV5-RSV-Fm showed better neutralizing activity, and there was a quantitative effect. The neutralizing antibody titer of mice immunized with rAAV5-RSV-Fm in the high-dose group reached 1:256 on day 120 (**Figure 3E**). Combined with the results of IgG detection by ELISA, these findings indicate that the vaccine could induce an effective antibody response.

### 3.4 Induction of cell-mediated immune responses by the rAAV5-RSV-Fm vaccine

**Figure 4:**
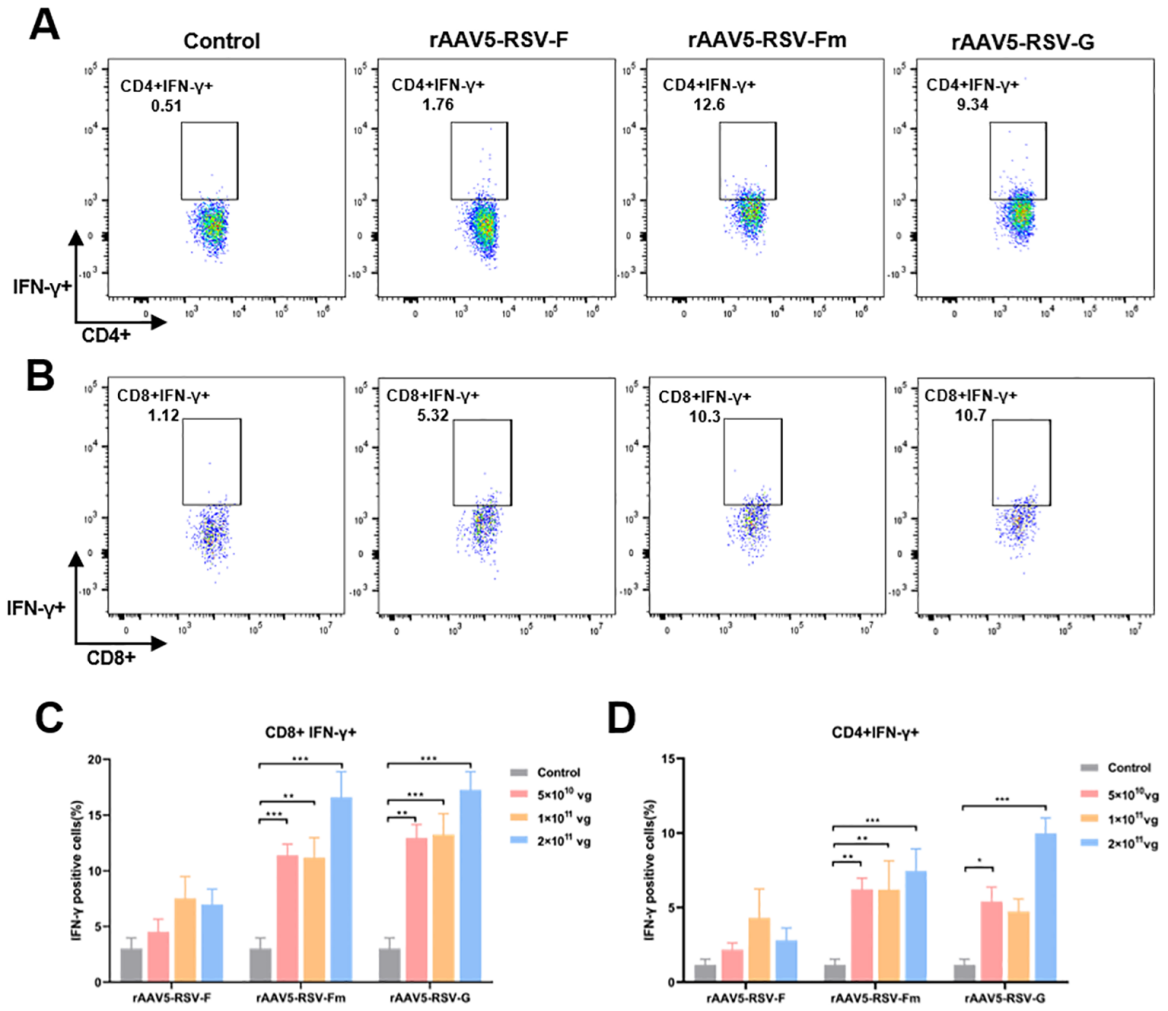
The rAAV5-RSV vaccine induced a protective cell-mediated immune response in mice at Day 75 after immunization. The rAAV5-RSV-Fm and rAAV5-RSV-G vaccines increase the IFN-γ levels in CD4+ **(A, D)** and CD8+ **(B, C)** T cells (n = 5 per group). Statistical significance was calculated via two-way ANOVA with Dunnett’s multiple comparisons. P values were adjusted for multiple comparisons. *p < 0.05, **p < 0.01, ***p < 0.001.

In the immune system, the levels of the antiviral factor interferon γ (IFN-γ) represent helper T-cell activity. To evaluate the cellular immunity of mice immunized with the rAAV5-RSV vaccine, we isolated peripheral blood mononuclear cells (PBMCs) from mice immunized with the rAAV5-RSV vaccine. Flow cytometry was used to analyze the expression of IFN-γ in CD4+ and CD8+ T cells (**Figure 4**). Cytokine IFN-γ secretion from CD4 cells and CD8 cells was upregulated compared to control after being immunized with the rAAV5-RSV-Fm and rAAV5-RSV-G vaccines. Moreover, the expression of IFN-γ in the high dose group was significantly greater than that in the low-dose group. The results of combination with neutralizing antibodies suggested that the rAAV5-RSV-Fm vaccine could effectively induce a powerful systemic humoral and cell-mediated immune response in mice.

### 3.5 Effective immunogenic responses elicited by intranasal administration of the rAAV5-RSV-Fm vaccine

For respiratory vaccines, early nasal IgA antibodies produced by mucosal immunization are the most abundant mucosal antibodies and play important roles in the effective neutralization of upper respiratory viruses. We selected the rAAV5-RSV-Fm vaccine expressing the pre-F construct for mucosal immunization, which showed good humoral immunity after intramuscular immunization. In the intramuscular immunization experiment described above, a dosage of 1×10^11^ vg demonstrated significant efficacy. Consequently, in anticipation of clinical application of the vaccine, dosages of 5×10^10^ vg and 1×10^11^ vg were selected for the experiment involving mucosal immunization.

**Figure 5:**
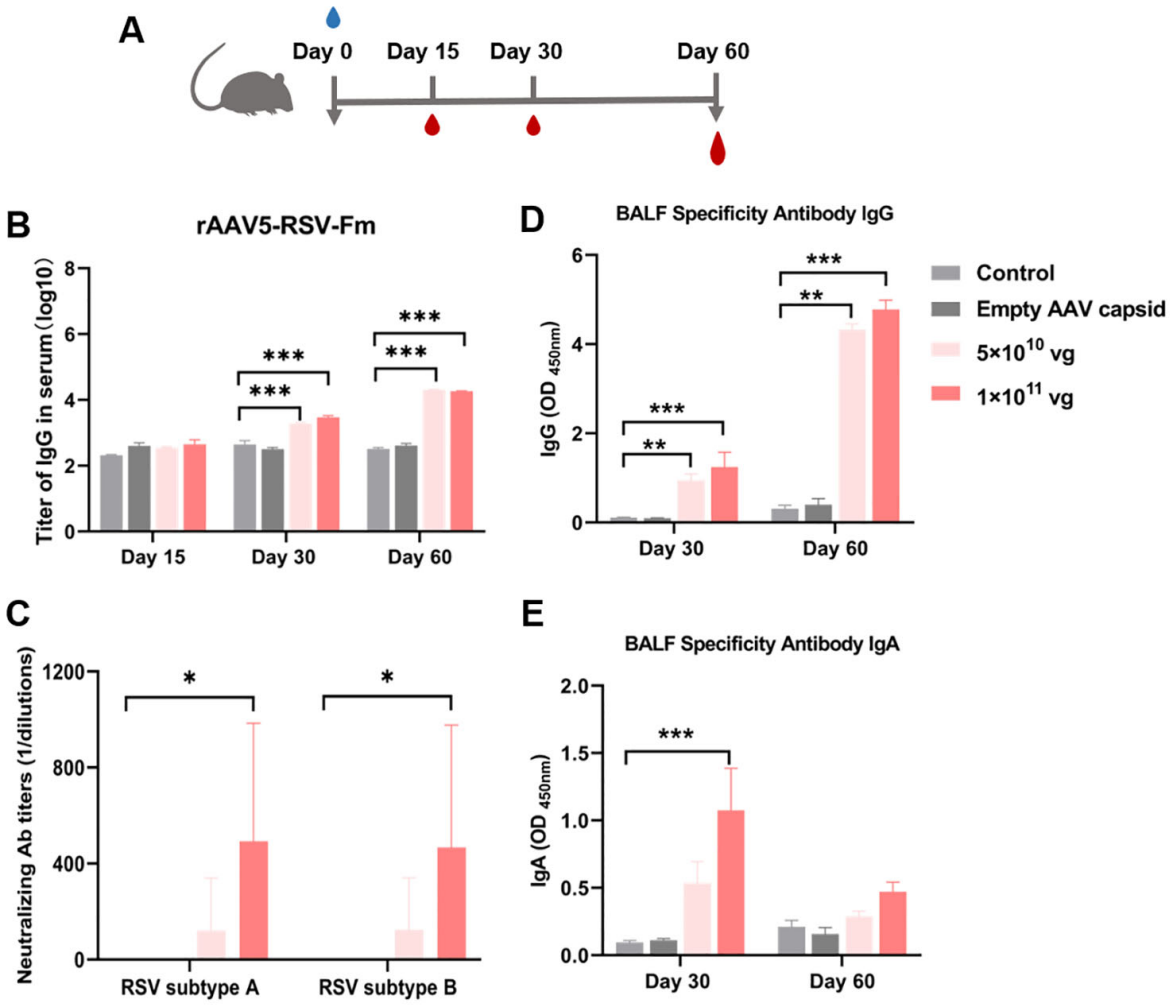
**(A)** Serum RSV-specific and neutralizing antibody titers induced by the recombinant AAV RSV-Fm vaccine. **(B)** Serum IgG titers at 15, 30 and 60 days after immunization were determined by ELISA. **(C)** Neutralizing activity against RSV subtypes A and B was assessed in 60 days after immunization. **(D, E)** Specific IgG and IgA antibodies in BALF from mice treated with recombinant AAV RSV-Fm were analyzed. The data are presented as the mean ± SD of antibody titer (n = 5 per group). Statistical significance was calculated via two-way ANOVA with Dunnett’s multiple comparisons **(B, D, E)**. P values were adjusted for multiple comparisons. Statistical significance was calculated via Kruskal-Wallis rank-sum test **(C)**. *p < 0.05, **p < 0.01, ***p < 0.001.

Blood samples were collected in 15, 30 and 60 days after vaccination (**Figure 5A**). The rAAV5-RSV-Fm vaccine increased the levels of specific IgG antibodies on day 60 after intranasal administration (**Figure 5B**). Sera samples obtained from mice 60 days after immunization effectively displayed elevated levels of neutralizing antibody titers against both RSV subtype A and subtype B strains. Specifically, mice immunized with rAAV5-RSV-Fm in the 1×10^11^ vg dose group exhibited a neutralizing antibody titer of 1:500 on day 60 (**Figure 5C**). These results suggest that the vaccine elicited a robust antibody response following mucosal immunization with a dosage of 1×10^11^ vg. On day 30 and 60, 5 mice of each group were sacrificed, and bronchoalveolar lavage fluid (BALF) was collected to determine specific IgG and IgA levels (**Figures 5D, E**). The levels of specific IgG and IgA at Day 30 in the lung lavage fluid of the vaccinated mice were significantly greater than those in the control and AAV shell groups, indicating that the immunized mice exhibited mucosal immunity.

## 4 Discussion

Viral vector vaccines, particularly those based on AAV vectors, have emerged as promising tools for eliciting robust immune responses against infectious diseases. The safety profile and ability to confer long-term antigen expression with just a single dose make the AAV vector platform highly attractive for vaccine development (31, 32). This platform ensures long-term expression of target antigens at high levels and provides stable immune effects with only a single dose immunization. Moreover, during out-breaks of new infectious diseases, the DNA sequence encoding pathogenic proteins can be swiftly replaced within this vaccine platform to rapidly produce vaccines for epidemic control. Our previous success in developing SARS-CoV-2 vaccines using the rAAV5 vector platform underscores its potential applicability in combating various infectious diseases (21). In this study, we extended our investigation to evaluate three rAAV5-RSV vaccine candidates that target RSV, with a focus on their efficacy in inducing neutralizing antibodies. Our preliminary results highlight the effectiveness of the rAAV5-RSV-Fm vaccine in eliciting high levels of neutralizing antibodies, thereby demonstrating the promising application of this adeno-associated viral vector vaccine platform in RSV vaccine development.

The RSV F and G proteins harbor distinct antigenic epitopes and have been the subject of numerous vaccine studies. While the G protein facilitates virion attachment to host cells (9), its role in inducing neutralizing antibodies remains limited. Our data indicate that the rAAV5-RSV-G vaccine expressing the full-length G protein elicits high levels of anti-G IgG antibodies in humoral immunity. However, neutralizing antibody tests suggest that it can-not neutralize viruses. This result is consistent with animal studies on vaccines targeting the G protein in which neutralizing antibodies were barely detectable but still provided protection against RSV infection in a mouse model (33, 34). The protective mechanism of the G protein may involve activating the complement system or anti-body-dependent cytotoxicity (ADCC) rather than neutralization. However, further investigations, including challenge experiments in animal models, are warranted to fully elucidate the protective efficacy of the rAAV5-RSV-G vaccine.

In contrast, the RSV F protein, with its various conformational states, is a more versatile antigenic target. Different vaccine modalities targeting the F protein have demonstrated varying degrees of success in inducing the production of neutralizing antibodies (35, 36). These vaccines contain either a pre-fusion F conformation or a post-fusion conformation. However, due to conformational changes that result in the loss of important epitopes such as site Ø, immune responses induced by the F protein differ between these two states. Multiple findings suggest that targeting the pre-fusion state is superior for eliciting neutralizing antibodies compared to targeting the post fusion state (22, 37). In this study, we selected two mutation sites (N67I and S215P) to stabilize the SC-DM (Pre-F) protein structure (22). Our study focused on stabilizing the pre-fusion conformation of the F protein, which is known to elicit potent neutralizing antibodies. For the first time, we employed the rAAV5 vector platform to deliver the Pre-F protein sequence and compared it with the original full-length F protein sequence. Our results showed successful binding of rAAV5-RSV-Fm vaccine-derived proteins with the D25 antibody but minimal binding with the 4D7 antibody, indicating successful stabilization of the Pre-F protein structure using the rAAV5 vector platform. Conversely, proteins derived from the rAAV5-RSV-F vaccine exhibited increased binding with the 4D7 antibody, suggesting the expression of more post-F conformation proteins. *In vivo* immunization studies at a dose of 2×10^11^ vg from day 15 to day 120 revealed that the rAAV5-RSV-Fm vaccine induced anti-F IgG antibodies, the levels of which peaked on day 60 and remained stable thereafter; additionally, high levels of neutralizing antibodies against both the RSV A and B strains were observed upon administration of the rAAV5-RSV-Fm vaccine, thus confirming its effectiveness in inducing the desired immunogenicity. In addition, the rAAV5-RSV-Fm vaccine was inoculated into mice intranasally to verify its mucosal immune effect. The results showed that the rAAV5-RSV-Fm vaccine could induce the protective antibodies by intramuscular or mucosal vaccination.

This research, however, is subject to several limitations. The limitations mainly exist in two aspects. Firstly, there is a constraint pertaining to the novelty of the RSV-Pre F protein configuration. The current inquiry concentrates on validating the feasibility of expressing the Pre-F protein configuration via the rAAV vector. Nonetheless, for sustained inquiry, the creation of an autonomously developed Pre-F protein configuration warrants exploration. Secondly, there exists a necessity for refinement in the validation experiments of immunogenicity concerning animal vaccines. Further investigation is warranted to delineate alterations in T-helper cell subtypes subsequent to vaccination, alongside the execution of challenge experiments to corroborate the protective efficacy of the rAAV5-RSV vaccine.

In conclusion, this study provides valuable information and techniques for further R&D of RSV vaccines using the rAAV5 vector platform. The distinct advantages of the rAAV5-RSV-Fm vaccine, which induces potent immune responses, underscore the potential application of this adeno-associated viral vector vaccine platform for combating RSV and other infectious diseases. Future research endeavors will focus on evaluating the protective efficacy of enhanced rAAV5-RSV-Pre F vaccines and elucidating their underlying mechanisms in protecting against RSV infection, thereby paving the way for the development of effective preventive strategies against this significant respiratory pathogen.

## Data Availability

The original contributions presented in the study are included in the article/**Supplementary Material**. Further inquiries can be directed to the corresponding authors.
