# Supplementary material for: Induction of neutralizing antibody responses by AAV5-based vaccine for respiratory syncytial virus in mice
Source: Front Immunol. 2024 Oct 14;15:1451433. doi: 10.3389/fimmu.2024.1451433 (PMC11513327; doi:10.3389/fimmu.2024.1451433)
Supplement: Supplementary file 1 [file DataSheet1.docx]

Supplementary Material

Induction of Neutralizing Antibody Responses by AAV5-based Vaccine for Respiratory Syncytial Virus in Mice

Gangyuan Ma #1, 2, Zeping Xu #3, Chinyu Li 3, Feng Zhou 1, Bobo Hu 1, Junwei Guo 3, Changwen Ke 6, Liqing Chen 1, Guilin Zhang 3, Hungyan Lau 3, Hudan Pan 4,5, Xixin Chen 3, Runze Li * 4,5, Liang Liu *1,4,5

*** Correspondence:**

Runze Li, Liang Liu
Runze Li* lirunzetk@163.com, Liang Liu* lliu@gzucm.edu.cn;

# Supplementary Figures

## Supplementary Figures


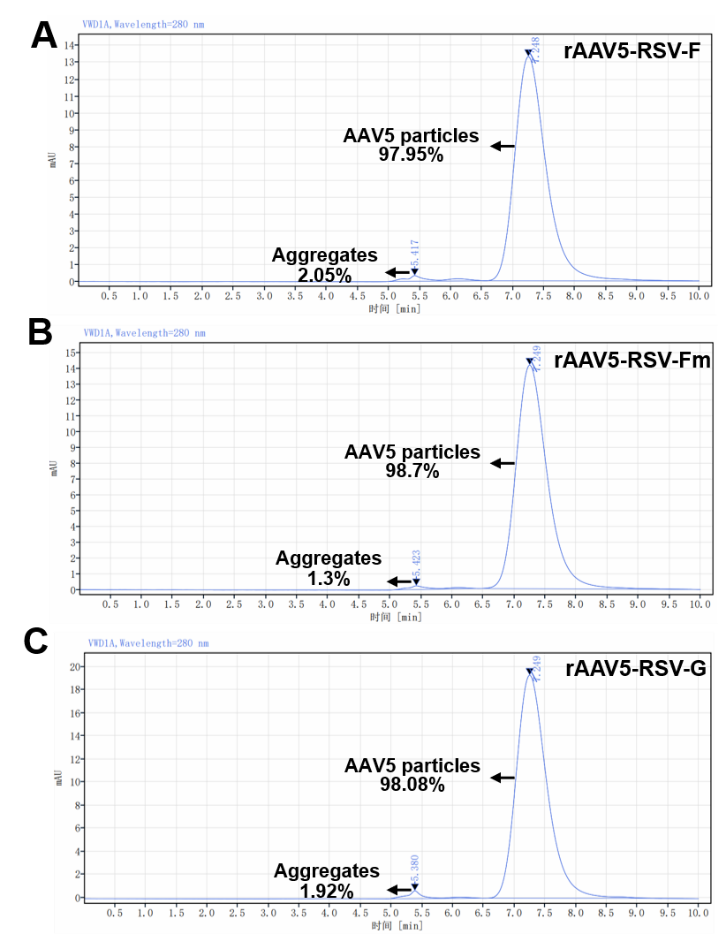


**Supplementary Figure 1.**

SEC-HPLC method and SRT SEC-500 column were used to detect the aggregates of rAAV5 vector, and the results of aggregates are shown in the Supplementary Figure S1. rAAV5-RSV-F, rAAV5-RSV-Fm and rAAV5-RSV-G vaccines could accurately isolate the aggregation peak (5.4min) and AAV5 particles peak (7.3min). The aggregates of the three rAAV5-RSV vaccines were 2.05%, 1.3% and 1.92%, respectively, which were all very low, indicating that the rAAV5 viral vector platform could produce highly purified rAAV vaccines.


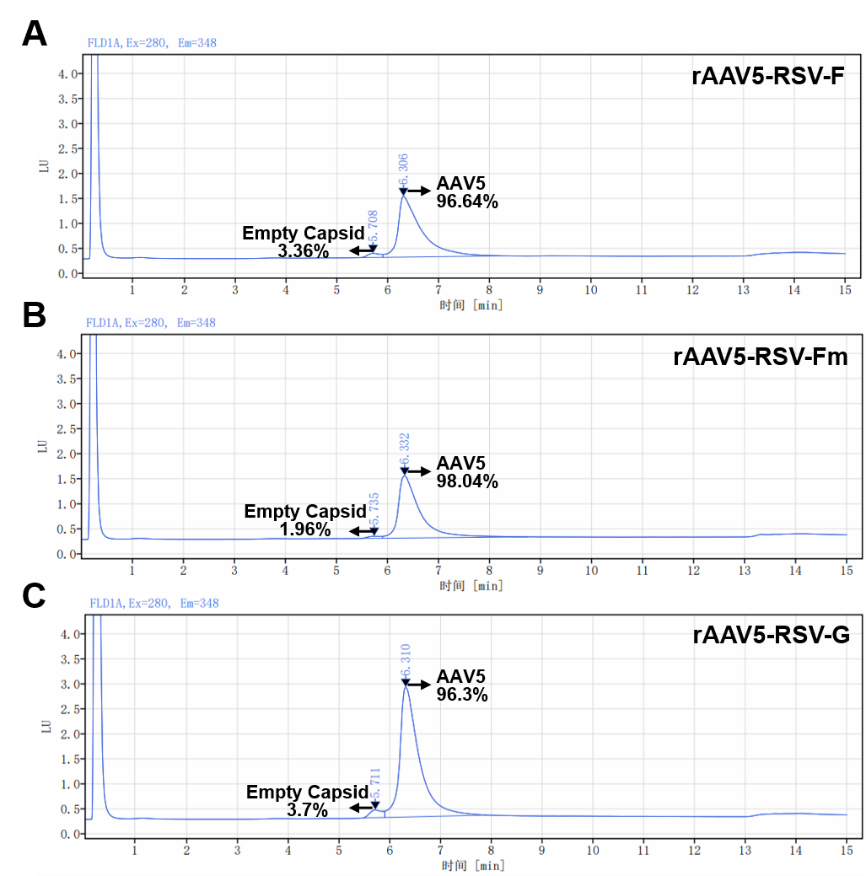


**Supplementary Figure 2.**

For the detection of the empty AAV capsid, the AEC-HPLC method and BIA /CIMac™ AAV empty/full - 0.1 Analytical Column were used to separate the empty capsid from the intact AAV5 vector using the AAV empty/full capsid analytical column. As shown in the Supplementary Figure S2, rAAV5-RSV-F, rAAV5-RSV-Fm, rAAV5-RSV-G vaccines all accurately separated the shell peak (5.7min) and the AAV5 complete vector solid peak (6.3min), and the rest had no stray peaks. The percentage of empty capsid of the three rAAV5-RSV vaccines were 3.36%, 1.96% and 3.7%, respectively, while the industry standard is less than 30%, suggesting that the constructed rAAV5 viral vector platform could produce AAV vaccines with low percentage of empty capsid and high purity.

**
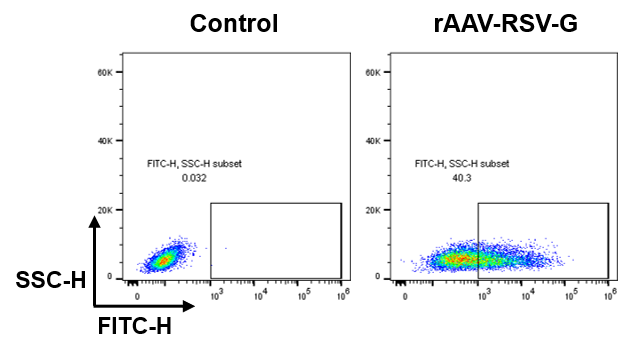
**

**Supplementary Figure 3.**

The target protein of RSV-G expressed by rAAV5-RSV-G vaccine was a membrane protein. Flow cytometry was also used to verify the correctness of RSV-G protein expressed by rAAV5-RSV-G vaccine. The 3D3 antibody that could accurately bind to the hRSV-G protein was used for verification. The Supplementary Figure 3 showed that compared with the control group, the target protein expressed by rAAV5-RSV-G vaccine had a binding positive rate of 40.3% with the 3D3 antibody, suggesting that it could correctly express the RSV-G protein.


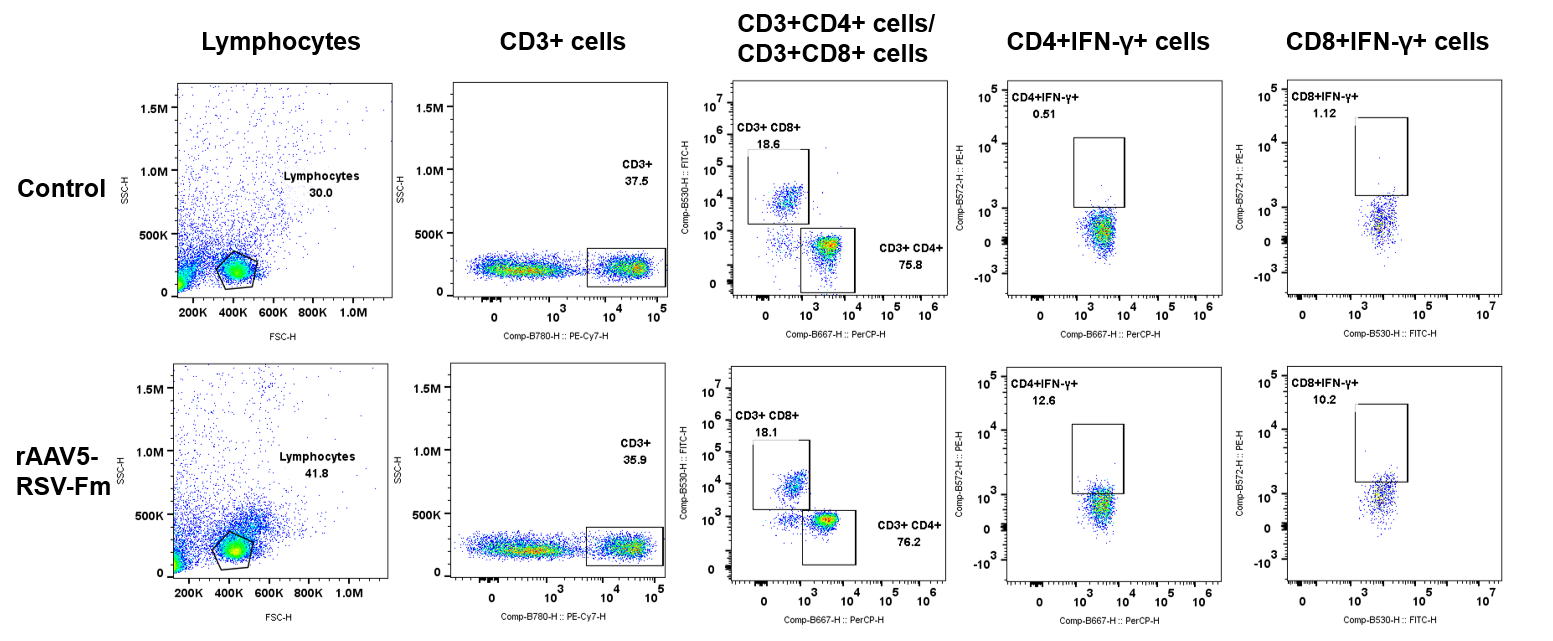


**Supplementary Figure 4.**

The flow cytometry gating strategy in Figure 4.
